# Exploitation of Apulian *Salicornia europaea* L. via NADES-UAE: Extraction, Antioxidant Activity and Antimicrobial Potential

**DOI:** 10.3390/molecules30163367

**Published:** 2025-08-13

**Authors:** Francesco Limongelli, Antonella Maria Aresta, Roberta Tardugno, Maria Lisa Clodoveo, Alexia Barbarossa, Alessia Carocci, Carlo Zambonin, Pasquale Crupi, Manuela Panić, Filomena Corbo, Ivana Radojčić Redovniković

**Affiliations:** 1 Department of Pharmacy-Drug Sciences, University of Bari Aldo Moro, Via E. Orabona 4, 70126 Bari, Italy; 2 Department of Biosciences, Biotechnology and Environment, University of Bari Aldo Moro, Via E. Orabona 4, 70126 Bari, Italy; 3 Department of Interdisciplinary Medicine (DIM), University of Bari Aldo Moro, Piazza Giulio Cesare 11, 70100 Bari, Italy; 4 Department of Agricultural, Food and Forest Science, University of Palermo, Viale delle Scienze, 90128 Palermo, Italy; 5 Faculty of Food Technology and Biotechnology, University of Zagreb, Pierottijeva 6, 10000 Zagreb, Croatia

**Keywords:** *Salicornia europaea* L., antioxidants, antimicrobial, polyphenols, natural deep eutectic solvents, ultrasound-assisted extraction

## Abstract

*Salicornia europaea* L. is a spontaneous halophytic plant, widespread in coastal environments, recognized for its high polyphenol content and bioactivities. In this study, a sustainable extraction strategy was developed by coupling natural deep eutectic solvents (NADESs) with ultrasound-assisted extraction (UAE) to recover bioactive compounds from autochthonous *S. europaea* collected in the Apulia region of southern Italy. Sixty-one NADES combinations were screened using COSMOtherm software, based on the predicted solubility of isorhamnetin, the major flavonol in *Salicornia* spp, to identify optimal hydrogen-bond donor (HBD) and acceptor (HBA) pairs. Six selected and prepared NADESs (B:CA, B:Suc, ChCl:U, ChCl:Xil, CA:Glc and Pro:MA) were used to extract *S. europaea*, and the resulting extracts were evaluated for total phenolic content (TPC), antioxidant capacity (DPPH, ABTS, FRAP) and antibacterial activity against four ATCC bacterial strains (*Enterococcus faecalis*, *Escherichia coli*, *Klebsiella pneumoniae* and *Staphylococcus aureus*). Among the tested extracts, Pro:MA exhibited the highest TPC (6.79 mg GAE/g) and interesting antioxidant activity (DPPH IC_50_ = 0.09 mg GAE/g; ABTS = 8.12 mg TE/g; FRAP = 2.41 mg TE/g). In the antibacterial assays, the Pro:MA extract demonstrated the highest activity, with minimum inhibitory concentrations (MICs) ranging from 0.1% to 0.4% *v*/*v* and minimum bactericidal concentrations (MBCs) from 0.2% to 0.8% *v*/*v*. In addition, the Pro:MA extract maintained TPC stability over a 90-day storage period. These findings support the NADES-UAE system as a green and efficient approach for the recovery of bioactive compounds and for the valorization of halophyte plants, such as *S. europaea*, with promising ready-to-use applications in the food, pharmaceutical and cosmeceutical sectors.

## 1. Introduction

*Salicornia* is a genus of succulent, salt-tolerant (halophytic), shrubby or herbaceous flowering plants that grow all year round in salt pans, beaches and mangroves, belonging to the *Amaranthaceae* family [1,2].

Although 64 *Salicornia* species (spp.) are reported, the most popular are native to North America (*S. bigelovii*), Europe (*S. europaea*, *S. ramosissima*, *S. fruticosa* and *S. herbacea*), Asia (*S. brachiata* and *S. herbacea*) and Africa (*S. arabica* and *S. fruticosa*) [1,2,3,4,5]. *Salicornia* spp. are also called glasswort, pickleweed, sea beans, sea asparagus or marsh samphire in America and Europe, while in Asia, they are known as umari keerai. Glasswort has a long history of diverse applications. For example, it has been harvested for food purposes, to extract salt, used as an additive in soap and glass production, and as a purifying, diuretic, analgesic and anti-inflammatory in folk medicine [1,2]. Many studies have shown that it may have antioxidant, anti-inflammatory and immunomodulatory activities, capable of counteracting the risk of obesity, cardiovascular disease and diabetes, along with potential applications in the treatment of constipation and cancer [1,2,6,7].

The key components of glasswort extracts, as characterized through various analytical techniques (e.g., HPLC-UV, HPLC-MS, GC-MS), include both primary metabolites, such as vitamins, amino acids, minerals, sterols and fatty acids, and secondary metabolites, including saponins, oxalates and polyphenols. The polyphenols present in *Salicornia* spp. primarily belong to the classes of phenolic acids, flavonoids and lignans [5,8,9,10,11]. Among phenolic acids, chlorogenic, tungtungmadic, ferulic, protocatechuic, caffeic, salicylic, syringic and coumaric acids are the most representative [7,12]. It has also been shown that it is possible to obtain flavonoids from *Salicornia* spp., such as isorhamnetin, quercetin, rutin, both aglycone and glycosylated, quercetin 3-O-β-D-glucopyranoside, isorhamnetin 3-O-β-D-glucopyranoside, noreugenin and isoquercitrin [1,11,13]. Among the lignans, the main isolated from *S. europaea* were syringaresinol 4-O-β-D-glucopyranoside, erythro-1-(4-O-β-D-glucopyranosyl-3,5-dimethoxyphenyl)-2-syringaresinoxyl-propane-1,3-diol, longifloroside B and acanthoside B [1,14].

Due to the positive biological effects of its constituents [15,16,17,18,19], there is a growing public interest in both the fresh and dry consumption of this vegetable and in the development of nutraceutical formulations, encouraging its cultivation and marketing. In Europe, *Salicornia* spp. is mainly cultivated in the Netherlands, Portugal and France [20]. In the Apulian region of southern Italy, although native species have long been recognized for multiple uses and as traditional food, the market is mainly based on the collection of wild plant stems [20]. Currently, to promote its marketing, research is underway and aimed at selecting genotypes and effective agri-food techniques to support its cultivation in the marginal marshy lands of the Puglia region. Along with this goal, the sustainable extraction of bioactive compounds from *Salicornia* spp. is a crucial step for their medical, nutraceutical and cosmeceutical uses. Extraction of polyphenols from glasswort generally involves conventional solid–liquid techniques, such as maceration in heated distilled water [21,22,23] for a short time (5 min) [22] or at room temperature with a longer extraction time (16–24 h) [21,23]. In the latter case, a greater recovery of phenolic compounds labile at high temperatures is guaranteed. Extraction with organic solvents alone or mixed to obtain different degrees of polarity can increase the yields of specific polyphenols from fresh or dried plants. For example, *S. europaea* ethanolic extracts are mainly rich in nine polyphenols, including chlorogenic acid, gallic acid, rutin and catechin [24]. *S. herbacea* with 80% acetone at room temperature for 24 h yields a high amount of pentadecyl ferulate [25]. To enhance the speed and yield of bioactive compound extraction from *Salicornia* spp., while also reducing processing times, thermal degradation losses, solvent and energy consumption, different advanced extraction techniques can be employed, including ultrasound-assisted extraction (UAE) [25,26,27,28,29], microwave-assisted extraction (MAE) [22,24,30], enzyme-assisted extraction (EAE) [31,32,33] and the extraction of supercritical fluids (SFEs) [34,35].

The increase in the world’s population, together with the overexploitation of natural resources, has made green and sustainable chemistry a collective effort aimed at overhauling chemical processes. The field aims to produce alternative solvents, catalysts and other ancillary chemicals that are less aggressive and safer for people and the environment than more traditional options [36]. In this context, natural deep eutectic solvents (NADESs) have emerged as a promising new subclass of deep eutectic solvents (DESs), characterized by higher biocompatibility, recyclability, cost-effectiveness, safety and adjustable physicochemical characteristics [36,37,38], making them ideal for use in the extraction of bioactive compounds from biomass [39,40]. NADESs are formed by mixing two or more components, represented by primary cellular metabolites, such as low-molecular-weight sugars, organic acids, alcohols, amines and amides [36,40]. Each component of the mixture acts as a hydrogen-bond donor (HBD) or acceptor (HBA). Their interaction during mixing lowers the melting point of each individual compound, resulting in a stable fluid system with distinct solubilizing characteristics. The driving force behind extraction is the participation of a wide range of phytocompounds in the formation of hydrogen bonds in a supramolecular network, which facilitates the extraction of a wide range of phytochemicals [37]. An important aspect of NADESs is their ability to be tailored for specific applications. By adjusting the ratio of HBDs and HBAs or adding additional components, such as water, the properties of NADESs, such as polarity, viscosity and solubility, can be fine-tuned. This flexibility allows for the efficient extraction of a diverse range of bioactive compounds, from polyphenols to alkaloids and essential oils. Moreover, NADESs have been shown to exhibit excellent solubilizing power for both hydrophilic and lipophilic compounds, which enhances their effectiveness in various extraction processes. Their low toxicity and biodegradability make them a highly attractive option in comparison to conventional organic solvents, particularly in industries where environmental impact is a critical concern [41]. According to the literature, NADES systems can improve the extraction of bioactive compounds with antimicrobial properties [42,43]. This is supported by previous studies confirming that NADESs exhibit antimicrobial properties and inhibitory effects toward different microorganisms, and antibacterial effects against both Gram-positive and Gram-negative bacteria. In addition, according to other studies, *S. europaea* polyphenol-rich extracts showed antimicrobial properties particularly against *S. aureus* and *E. coli*, highlighting the antimicrobial potential of polyphenol-rich halophyte plants [42,44,45].

This study presents an innovative approach for valuing autochthonous Apulian *S. europaea* through the application of ecological and sustainable practices, exploring for the first time the selection of NADESs through a software-based predictive approach, based on the simulation of flavonoid secondary metabolites. Isorhamnetin was selected, being the most representative polyphenol class in *Salicornia* spp. [1,46,47,48]. The NADES-UAE extracts obtained using selected HBAs and HBDs with the software-based predictive approach, characterized in terms of total antioxidant contents, microbiological activities and stability over time, could potentially be ready-to-use in food, nutraceutical, pharmaceutical and cosmeceutical fields without subsequent purification steps.

## 2. Results and Discussion

### 2.1. NADES Screening Using COSMOtherm

The process of identifying the optimal NADESs for the extraction of specific bioactive compounds is a crucial step and can be time-consuming and intensive [41]. COSMOtherm software was employed to screen NADESs to select the most promising solvent for the extraction of antioxidant polyphenolic compounds from *S. europaea* stems. The first step was to evaluate the solubility of the flavonol isorhamnetin, as the most representative polyphenol class present in *S. europaea* [1]. NADESs with two components in different molar ratios using the COSMOtherm option-activity coefficient calculation function were evaluated. To rationally guide the experimental selection of NADESs for isorhamnetin solubilization, the screening included 61 different NADES combinations from our internal database, incorporating various HBDs, such as polyols, sugars, amino acids and organic acids, paired with common HBAs, like choline chloride and betaine. This broad chemical space allowed us to identify trends in solubility behavior based on the calculated logarithmic activity coefficients (ln γ) for isorhamnetin. The calculation of the activity coefficient (ln γ) was performed using 30% water to decrease viscosity, since high viscosities in NADESs can limit extraction efficiency [41]. The computed ln(γ) values across the 61 NADESs are reported in Table 1, where lower values indicate stronger solubilization potential.

As shown in Table 1, betaine-based NADESs show an enhanced affinity for isorhamnetin, particularly when combined with glucose, sucrose, glycerol or malic acid. In contrast, choline chloride-based NADESs and those composed solely of amino acids, organic acids or polyols generally show weaker interactions. For example, betaine:ethylene glycol (1:2) yielded the lowest ln γ value (−3.47), followed by betaine:sucrose (4:1; −3.88) and choline chloride:ethylene glycol (2:1; −4.79). While these values suggest high solubilization potential, practical considerations, such as viscosity, toxicity and biocompatibility, were also considered. Based on this screening, a subset of NADESs was selected for the experimental evaluation. The final selection included B:CA, B:Suc, ChCl:U, ChCl:Xyl, CA:Glc and Pro:Ma, representing a chemically diverse group of systems with components that are food-grade or biocompatible. These combinations were chosen to reflect a range of solvation behaviors and chemical functionalities while ensuring relevance for potential applications.

### 2.2. TPC, Antioxidant and Antiradical Assays

**Figure 1:**
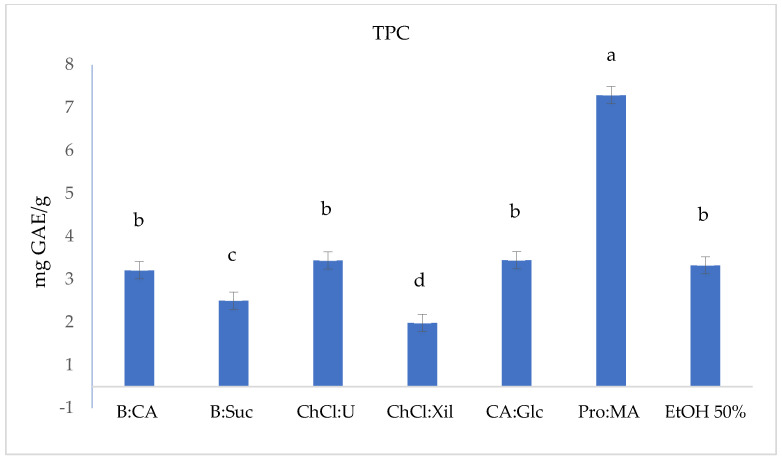
Total phenolic content (TPC, mg GAE/g) in *S. europaea* NADES (6) and EtOH 50% conventional extracts. TPC values are expressed as the means (*n* = 3). Different letters within a column indicate significant differences between samples at *p* < 0.05.

**Figure 2:**
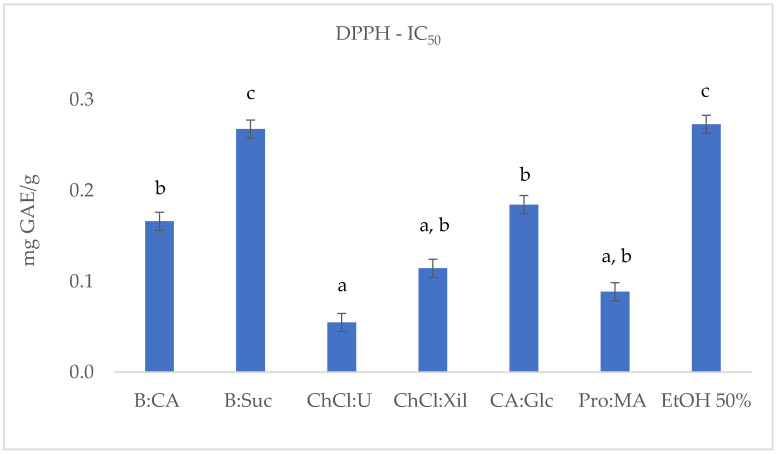
DPPH (IC_50_ mg GAE/g) results of *S. europaea* NADES-UAE (6) and conventional EtOH 50% UAE (1) extracts. IC_50_ is expressed as the means (*n* = 3). Different letters within a column indicate significant differences between samples at *p* < 0.05.

**Figure 3:**
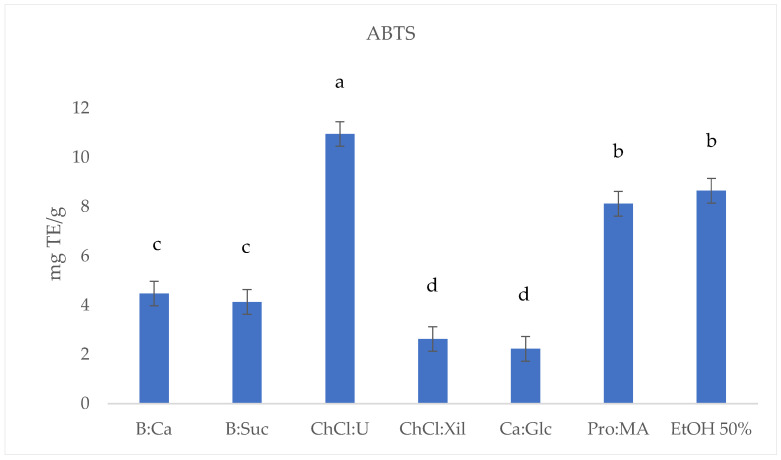
ABTS (mg TE/g) results of *S. europaea* NADES-UAE (6) and conventional EtOH 50% UAE (1) extracts. Values are expressed as the means (*n* = 3). Different letters within a column indicate significant differences between samples at *p* < 0.05.

**Figure 4:**
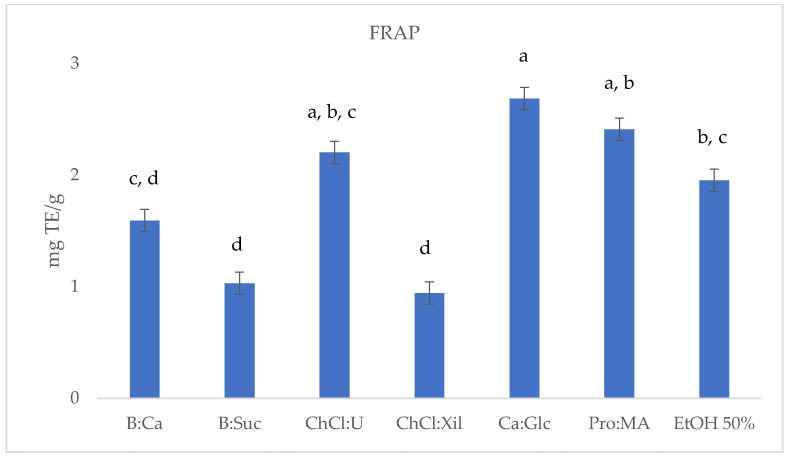
FRAP (mg TE/g) results of *S. europaea* NADES-UAE (6) and conventional EtOH 50% UAE (1) extracts. Values are expressed as the means ± SD (*n* = 3). Different letters within a column indicate significant differences between samples at *p* < 0.05.

To evaluate the extraction efficiency of the selected NADESs for phenolic antioxidant compounds from *S. europaea* stems, the TPC, DPPH, ABTS and FRAP were determined. The results are reported in Table 2 and Figure 1, Figure 2, Figure 3 and Figure 4.

The TPC in the *S. europaea* NADES-UAE extracts and hydroalcoholic UAE extract (EtOH 50%) as conventional extraction was measured using the F-C assay, and the results are shown in Table 2 and Figure 1. The TPC, expressed in mg GAE/g of dry *S. europaea* (Figure 1), varies significantly across the tested NADESs and conventional hydroalcoholic extract (EtOH 50%). Pro:MA, with the highest TPC value (6.79 ± 0.007 mg GAE/g), showed to be the most promising NADES-UAE extract among those tested, followed by CA:Glc, ChCl:U and EtOH 50%, all of which showed average TPC values in the range of 2.94–2.82 mg GAE/g. In contrast, ChCl:Xil and B:Suc showed lower TPC values, with values of 1.48 ± 0.007 mg GAE/g and 2.0 ± 0.001 mg GAE/g, respectively.

Pro:MA NADES exhibits both a relatively low viscosity (0.0150 Pa·s) and an acidic pH of 2.67, which likely contribute to its enhanced extraction performance by promoting better mass transfer and increased solubility of phenolic compounds. These attributes, combined with Pro:MA’s polarity (50.19 ENR kcal·mol^−1^), support its superior TPC yield. These physicochemical properties are consistent with the findings reported by Panić et al. (2019) [49], where Pro:MA was highlighted as a highly effective NADES for phenolic extraction from grape pomace [49]. Notably, solvent systems with lower predicted ln γ values, such as B:Scu and B:CA, also demonstrated a higher phenolic content, suggesting a correlation between theoretical solvation favorability and experimental extraction efficiency. This alignment supports the reliability of COSMO-RS predictions in guiding solvent selection for optimized isorhamnetin extraction [49,50,51].

The DPPH test was performed for the six *S. europaea* NADES-UAE extracts and for the conventional *S. europaea* UAE ethanolic extract (EtOH:H_2_O, 1:1). As shown in Table 2 and Figure 2, the IC_50_ values, indicating the concentration required to scavenge 50% of free radicals, are generally lower for most NADES-UAE extracts compared to the conventional 50% EtOH extract. The only exception was the B:Suc extract, whose IC_50_ value (0.27 ± 0.017 mg GAE/g) was comparable to that of the hydroalcoholic mixture. The ChCl:U extract showed the highest potential with its lowest IC_50_ value (0.05 ± 0.009 mg/mL), followed by Pro:MA (0.09 ± 0.003 mg GAE/g). ChCl:Xil (0.11 ± 0.012 mg GAE/g) was slightly higher than ChCl:U, followed by CA:Glc (0.18 ± 0.023 mg GAE/g) and B:CA (0.17 ± 0.019 mg GAE/g) extracts.

The ABTS assay results (Figure 3) reveal a considerable variability in antioxidant activity across the *S. europaea* NADES-UAE extracts. ChCl:U (10.96 ± 0.010 mg TE/g) demonstrated the highest antioxidant values, followed by conventional EtOH 50% *S. europaea* extract (8.65 ± 0.009 mg TE/g) and Pro:MA (8.12 ± 0.008 mg TE/g). B:CA (4.48 ± 0.003 mg TE/g) and B:Suc (4.13 ± 0.006 mg TE/g) exhibited moderate activity, while ChCl:Xil (2.63 ± 0.007 mg TE/g) and CA:Glc (2.23 ± 0.011 mg TE/g) showed the lowest activity among the extracts. NADES-UAE extracts, such as ChCl:U and Pro:MA, appear to possess higher antioxidant activities compared to the conventional ethanolic UAE extract (Table 2, Figure 3).

The FRAP assay results reported in Table 2 and Figure 4 indicate varying antioxidant capacities among the *S. europaea* NADES-UAE extracts investigated in this study. CA:Glc showed the highest antioxidant activity with a FRAP value of 2.69 ± 0.032 mg TE/g dry, followed by Pro:MA (2.41 ± 0.017 mg TE/g) and ChCl:U (2.20 ± 0.008 mg TE/g), all demonstrating interesting antioxidant activities. B:CA (1.59 ± 0.008 mg TE/g) and EtOH 50% (1.95 ± 0.003 mg TE/g) displayed moderate antioxidant activity, while B:Sac (1.03 ± 0.002 mg TE/g) and ChCl:Xil (0.98 ± 0.008 mg TE/g) showed the lowest activity. These findings suggest that certain NADES-UAE extracts may offer enhanced antioxidant properties compared to conventional *S. europaea* ethanolic UAE extracts.

The antioxidant profiles of *S. europaea* extracts obtained via NADES-UAE and 50% EtOH-UAE revealed that Pro:MA and CA:Glc exhibited the most promising results across most assays, exhibiting high TPC values and considerable antioxidant activities, as evidenced by their higher values in the DPPH, FRAP and ABTS results. ChCl:U showed antioxidant potential particularly in DPPH and ABTS assays. B:CA and EtOH 50% yield moderate results in the various assays, with EtOH 50% exhibiting the weakest DPPH radical scavenging activity value among the samples, while ChCl:Xil consistently underachieves across all assays, indicating its relatively low antioxidant potential. Pearson’s linear correlation analysis was performed among the computed solubilization potential ln(γ) values of the six selected NADESs (Table 1) and the TPC and antioxidant bioactivity values (DPPH, ABTS, FRAP) (Table 2). A strong correlation was observed between ln(γ) and the FRAP assay values (r = 0.79), and a moderate correlation was found between ln(γ) and the TPC values (r = 0.46). However, no significant correlation was observed between ln(γ) and the results obtained from the DPPH assay (r = −0.20) or the ABTS assay (r = −0.11).

The ability of NADESs to effectively solubilize and stabilize phenolic compounds may contribute to the observed moderate correlation between ln(γ) and TPC, and the strong correlation between ln(γ) and FRAP values. Indeed, the antioxidant activity of phenolic compounds is due to the presence of hydroxyl groups and their ability to donate an electron or hydrogen atom to free radicals, as well as to act as metal cation chelators and singlet oxygen quenchers, properties relevant to the FRAP assay’s mechanism. On the other hand, the absence of significant correlations with the DPPH and ABTS assays may be due to their intrinsic reaction mechanisms and specific molecular interactions, which may be less dependent on the solubilization potential of the NADES systems and the concentration of solubilized phenolic compounds.

### 2.3. Antibacterial Studies

In order to evaluate the bioactivity of *S. europaea* extracts obtained using NADES-UAE and 50% EtOH-UAE, in vitro antibacterial assays were also performed. Indeed, recent studies demonstrated that extracts of *S. europaea* exhibit strong antibacterial effects against both Gram-positive and Gram-negative bacteria due to the presence of several bioactive compounds, including polyphenols [42]. This effect was investigated by means of the microdilution method following the Clinical and Laboratory Standards Institute (CSLI) methodology against four bacterial strains belonging to the American Type Culture Collection: *E. faecalis* 29212 (Gram-positive), *S. aureus* 29213 (Gram-positive), *E. coli* 25922 (Gram-negative) and *K. pneumoniae* 13883 (Gram-negative). Considering that previous studies documented the inhibitory effects of NADESs toward different microorganisms [43], we analyzed, in parallel, the activity of pure NADES systems (B:CA, B:Suc, ChCl:U, ChCl:Xil, CA:Glc, Pro:MA) and 50% EtOH in the same dilution range applied for the *S. europaea* extracts. Levofloxacin, a well-known broad-spectrum fluoroquinolone antibiotic, was used as a reference due to its established efficacy against both Gram-positive and Gram-negative bacteria. Its inclusion ensures the reliability and validity of the test by providing a reference for assessing the antimicrobial activity of the extracts.

Table 3 and Table 4 report the obtained data expressed as minimum inhibitory concentration (MIC) % *v*/*v*, defined as the lowest concentration of samples where no growth is observed, and minimum bactericidal concentration (MBC) % *v*/*v*, defined as the lowest concentration of the compound at which no visible bacterial growth was observed on the agar plates, indicating that ≥99.9% of the initial bacterial population had been killed. In particular, Table 3 reports the data obtained for NADES systems and EtOH 50%, themselves, whereas Table 4 reports the results obtained with *S. europaea* NADES-UAE and EtOH-UAE extracts.

As regards the *S. europaea* NADES-UAE extracts, the results reveal some differences in antimicrobial efficacy depending on the extract and bacterial strain tested. Our findings reveal that, among the NADESs tested, CA:Glc possess the highest activity against all tested bacterial strains. The results, expressed as MIC, demonstrate a substantial variation in antimicrobial efficacy depending on the solvent system used for extraction. Among the tested extracts, the one obtained using Pro:MA as the extraction solvent showed the most interesting antibacterial activity, with MIC values ranging from 0.1% to 0.4% *v*/*v* and MBC values from 0.2% to 0.8% *v*/*v*. This indicates a strong inhibitory and bactericidal effect against all tested strains on both Gram-positive and Gram-negative bacteria. Notably, this extract showed a remarkable improvement in antibacterial activity compared to the NADES itself, which exhibited MIC values between 3.1% and 25% *v*/*v* and MBC values between 6.3% and 50% *v*/*v*, demonstrating the antimicrobial activity of the *S. europaea* extract, particularly when extracted from Pro:MA. When considering the MIC values, this corresponds approximately to a fold reduction ranging from 31- to 62-fold, highlighting a strong synergistic effect between the NADESs and the bioactive compounds extracted from *S. europaea.* The most pronounced activity was observed against the Gram-positive strains *E. faecalis* and *S. aureus* with MICs amounting to 0.1% *v*/*v*. However, the activity observed against the Gram-negative strains, such as *E. coli* and *K. pneumoniae*, is also noteworthy since it differs only slightly from the effect observed against Gram-positive strains. Indeed, this result is particularly interesting since both bacterial species represent opportunistic pathogens with a high level of intrinsic antibiotic resistance due to their low outer membrane permeability and active efflux mechanisms.

B:CA and CA:Glc extracts also displayed noteworthy antimicrobial activity, with MIC values ranging from 0.2% to 0.4% *v*/*v*. These extracts demonstrated good efficacy against all tested strains, with a slight preference for Gram-positive bacteria. Interestingly, B:CA retained an interesting effectiveness, even against Gram-negative bacteria. Compared to the solvent alone, which exhibited MIC values of 1.6% *v*/*v* for *E. faecalis*, *S. aureus* and *E. coli*, and 25% *v*/*v* for *K. pneumoniae*, the extracts showed a remarkable enhancement in antibacterial activity, particularly against *K. pneumoniae*, where the MIC value dropped from 25% *v*/*v* to just 0.4% *v*/*v*. This corresponds to an approximate fold reduction of 4x for *E. faecalis, S. aureus* and *E. coli*, and a 62.5-fold reduction for *K. pneumoniae.* Similarly, CA:Glc displayed a significant improvement in activity, as its MIC values against Gram-positive and Gram-negative bacteria were reduced to a range of 0.2–0.4% *v*/*v* when compared to the solvent alone, which required concentrations of at least 1.6–3.1% *v*/*v*. This corresponds to an approximate fold reduction ranging from 4× to 15.5×, further supporting the enhanced antibacterial effect of the extract compared to the pure NADESs. This highlights the role of bioactive compounds extracted by the NADES system. Conversely, extracts obtained using B:Suc, ChCl:U and ChCl:Xil exhibited weaker antibacterial activity, with MIC values of 25% *v*/*v* or higher against most tested bacteria. The lowest efficacy was observed for B:Suc and ChCl:Xil, particularly against *E. coli* and *K. pneumoniae*, where MIC values exceeded 50% *v*/*v*. This indicates that these solvent systems may not be optimal for extracting antibacterial compounds from *S. europaea*. Compared to their respective solvents alone, which also displayed high MIC values (≥25% *v*/*v*), the extracts did not demonstrate a notable improvement in antibacterial activity. In particular, ChCl:U showed no significant reduction in MIC values after extraction, maintaining MICs of 25% *v*/*v* against *E. faecalis* and *S. aureus* and exceeding 50% *v*/*v* for *E. coli* and *K. pneumoniae*. The ethanol–water extract (EtOH 50%) demonstrated moderate activity, with MIC values ranging from 3.1% to 12.5% *v*/*v*, showing better efficacy against Gram-positive bacteria compared to Gram-negative strains. While it is less effective than the best-performing NADES extracts, it still exhibited superior activity compared to the least effective NADES formulations. Notably, when compared to the ethanol–water solvent alone, which displayed MIC values in the range of 12.5–25% *v*/*v* for *S. aureus* and *E. faecalis* and 25% *v*/*v* for *E. coli* and *K. pneumoniae*, the extract showed a considerable enhancement in antibacterial activity, particularly against Gram-positive bacteria.

Regarding MBC values, an interesting finding is that they differed by only one dilution factor compared to MIC values for all tested extracts. Indeed, in all cases, the MBC/MIC ratio was ≤2, indicating a bactericidal effect according to CLSI definitions. This suggests that the extracts not only inhibit bacterial growth at low concentrations but also possess bactericidal properties at slightly higher concentrations.

These results highlight the effectiveness of NADESs in enhancing the extraction of bioactive compounds with antibacterial properties from *S. europaea*, particularly when using Pro:MA, B:CA, and CA:Glc as solvents. The significant differences in MIC values between extracts suggest that solvent selection plays a crucial role in optimizing the antibacterial potential of plant-derived compounds.

The antibacterial activity observed in *S. europaea* extracts obtained with NADESs aligns with previous studies highlighting the antimicrobial potential of polyphenol-rich halophyte plants. Several reports have demonstrated that polyphenol-rich extracts from *S. europaea* exhibit antibacterial properties, particularly against *S. aureus* and *E. coli* [4]. The present study further confirms these findings, showing that extracts obtained using Pro:MA, B:CA and CA:Glc displayed strong inhibitory effects against both Gram-positive and Gram-negative bacteria, with MIC values as low as 0.2% *v*/*v*. Furthermore, the antibacterial efficacy of NADES-based extracts was superior to that of the 50% ethanol extract, which exhibited MIC values ranging from 3.1% to 12.5% *v*/*v*. This is consistent with studies suggesting that NADESs can enhance the extraction and stability of bioactive compounds with antimicrobial properties [42] since the results also confirm previous findings that NADESs themselves possess antimicrobial properties, as seen in the MIC and MBC values of the solvent controls [52]. These findings indicate that the NADES-based extraction system is capable of more efficiently extracting bioactive compounds. The observed enhancement of bioactivity could also result from NADES-induced supramolecular network formation, which may lead to conformational changes or stabilization of phenolics compounds [50,51,52]. However, it cannot be excluded that the NADES itself may contribute to the observed antimicrobial activity. In addition, several studies have shown that NADESs composed of naturally occurring metabolites, such as proline, malic acid, betaine and citric acid, generally exhibit low-to-moderate cytotoxicity, particularly at the dilution levels typically used in antimicrobial assays [53,54,55]. These findings suggest that the concentrations at which antimicrobial activity was observed in our study are unlikely to pose significant cytotoxic risks. The results of this study suggest that the NADES-UAE approach could be a valuable method for obtaining antimicrobial compounds from *S. europaea*, potentially expanding its applications in pharmaceutical, food preservation and cosmeceutical sectors.

### 2.4. TPC Stability Over Time (Extracts)

**Figure 5:**
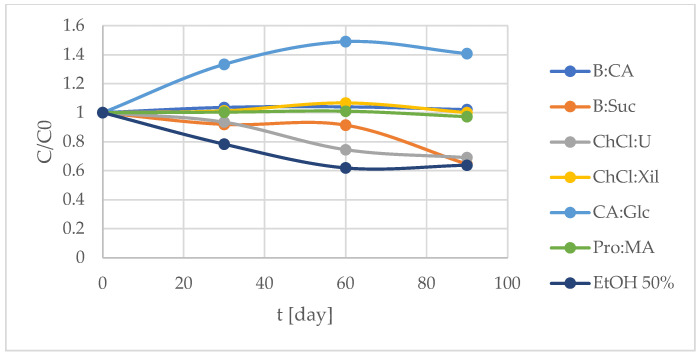
Degradation curve of total phenolic content (TPC) monitored over 90 days at 25 °C ± 2 °C. Results are expressed as the means (*n* = 3).

The TPC values over a 90-day period are presented in Table 5 and Figure 5, highlighting some *S. europaea* NADES-UAE extracts in preserving total phenolic content and related antioxidant activity. The most stable extracts, in terms of maintaining TPC over time, were *S. europaea* NADES-UAE Pro:MA, B:CA and Ch:Cl systems. Pro:MA showed the highest TPC values, followed by B:CA and ChCl:Xil. In contrast, B:Suc, ChCl:U and particularly 50% EtOH extracts exhibited the highest variability since their TPC values decreased over the 90-day storage period (Table 5, Figure 5).

These findings align with previous reports indicating that NADESs can enhance the stability of bioactive compounds compared to conventional solvents, like ethanol. This enhanced stability is often attributed to the strong hydrogen bonding and supramolecular interactions between polyphenolic compounds and NADES constituents, which reduce the mobility of solutes and limit their exposure to oxidative conditions [56]. In particular, NADESs composed of proline or citric acid with sugar-based components have shown promising results in protecting phenolic compounds during storage [50]. The stability of extracted antioxidant compounds is a crucial factor when selecting an appropriate extraction methodology, especially for applications in the food, pharmaceutical and cosmetic industries. Stability assessment involves monitoring the preservation of antioxidant capacity and phenolic content over time, particularly under storage conditions. NADESs have demonstrated potential not only as efficient green extraction media, but also as stabilizing agents for sensitive bioactive compounds. Their intrinsic antioxidant properties, along with their high viscosity, strong hydrogen bonding networks and antimicrobial activities, contribute to reduced molecular mobility and limited TPC decrease during storage [56]. This protective effect enhances the long-term stability of phenolic compounds amounts compared to conventional solvents.

## 3. Materials and Methods

### 3.1. Chemicals and Reagents

Betaine (B), choline chloride (ChCl), citric acid (CA), ethanol (EtOH), glucose (Glc), malic acid (MA), proline (Pro), sucrose (Suc), urea (U), ultrapure water (H_2_O) and xylitol (Xil) were acquired from Merck Life Science S.r.l. (Milan, Italy). 2,2-Diphenyl-1-picrylhydrazyl (DPPH), Folin–Ciocalteu reagent (F-C), 2,4,6-tri(2-pyridyl)-S-triazine (TPTZ), gallic acid (GA), Levofloxacin, Trolox (T), ethanol (EtOH), methanol (MeOH), sodium carbonate (Na_2_CO_3_), potassium persulfate (K_2_S_2_O_8_), sodium acetate (CH_3_COONa), ferric chloride (FeCl_3_) and acid solutions were purchased from Sigma-Aldrich (Milan, Italy). The ammonium salt 2,2′-azinobis (3-ethylbenzothiazolin-6-sulfonic acid) (ABTS) was obtained from Alfa Aesar (Karlsruhe, Germany). Mueller–Hinton Broth was acquired from Oxoid, Milan, Italy.

### 3.2. Plant Material

Fresh *Salicornia europaea* L. succulent green stems were collected from the Apulian Adriatic coast (40°59′41.64″ N 17°13′21.14″ E) in June 2023 (Voucher specimen: SE-0623-001). The freshly harvested stems were washed with water and gently dried at 35 °C for 48 h. Subsequently, the dried plant material was ground into a fine powder using an IKA A11 base homogenizer (IKA^®^-Werke GmbH & Co., Staufen, Germany); then, the powder was sieved (Endecotts Ltd., London, UK) to obtain a homogeneous particle size with particles approximately 200 μm in diameter, stored at −20 °C before extraction as described later.

### 3.3. Bacterial Strains

Bacterial strains from the American Type Culture Collection (ATCC, Rockville, MD, USA), *E. faecalis* ATCC 29212, *S. aureus* ATCC 29213, *E. coli* ATCC 25922 and *K. pneumoniae* ATCC 13883, were used as freeze-dried discs.

### 3.4. COSMO Simulation

For the COSMO simulation, the first step involved geometry optimization and molecules density calculation using discrete Fourier transform (DFT). Each molecule was optimized using the COSMO-BP-TZVP template from the TURBOMOLE software package. The optimization involved the def-TZVP basis set, DFT calculations with the B-P83 functional at the level of theory and the COSMO-RS solvation model with infinite permittivity. The optimization process was conducted to ensure the accurate structural representation of the molecules, which is essential for subsequent COSMO calculations. All calculations were executed using BIOVIA COSMOtherm 2020 (version 20.0.0). For this study, choline chloride salts, which were applied as hydrogen-bond acceptors (HBAs), were treated as ion pairs. Organic acids were considered in their protonated form. The NADESs were modeled as binary mixtures of hydrogen-bond donors (HBDs) and HBAs, with a fixed stoichiometric ratio. The COSMO calculations were performed to predict the activity coefficients (ln(γ)) of the target compound isorhamnetin derivatives since they are among the most represented polyphenol class of *Salicornia* spp. hydroalcholic extracts [46,48]. For hydrophilic NADESs, 30% water was added, while for hydrophobic DESs, no water was included. The output of the calculations provided the activity coefficients for the compounds at infinite dilution and 50 °C, which were used to determine the solubility behavior in the different solvents. Then, COSMOtherm 2020 software was used as a tool to predict the activity coefficient of isorhamnetin and quercetin in NADESs at 50 °C with the addition of 30% water.

### 3.5. NADES Preparation

NADESs were prepared as described by Karakula and coworkers [57]. Briefly, HBAs and HBDs were mixed in flasks at specified molar ratios as shown in Table 6; then, 30% of water was added (*v*/*v*). Mixtures were stirred at 50 °C for 2 h until transparent liquids formed [41]. NADESs were stored sealed at room temperature until use. NADES composition, including HBA, HBD, molar ratios and acronyms, are summarized in Table 6.

### 3.6. NADES-UAE Extraction

For the extraction, 1.5 g of *S. europaea* powder was mixed with 15 mL of NADESs (Table 6). Extractions were performed in an ultrasonic bath (Elmasonic P30H, Elma Schmidbauer GmbH, Singen, Germany) at a power of 100 W and frequency of 38 kHz, at 30 °C ± 5 °C for 30 min [58,59]. For comparison, a hydroalcoholic UAE was conducted using 1.5 g of *S. europaea* in 15 mL of EtOH:H_2_0 50% (1:1, *v*/*v*) under identical conditions adopted for NADESs [41].

### 3.7. Determination of Total Polyphenol Content (TPC)

The total phenolic content (TPC) was evaluated using the Folin–Ciocalteau (F-C) assay on a microplate reader (Tecan Infinite Pro 200 Microplate Reader, Männedorf, Switzerland) using spectrophotometric detection and 96-well microtiter plates (Greiner 96 Flat Bottom Transparent Polystyrene) [60]. A series of gallic acid (GA) standard solutions at different concentrations (0.025–0.25 mg/mL) was prepared and used to construct a calibration curve (y = 5.8893x + 0.0224, R^2^ = 0.9992). Briefly, 12.5 µL of GA standard solution or *S. europaea* extract was placed in each well followed by 12.5 µL of MeOH, 12.5 µL of F–C reagent and 163 µL of distilled water. After 5 min, 50 µL of Na_2_CO_3_ solution (20%) was added to each well, and the mixture was incubated to react for 90 min at 30 °C. Then, the absorbance was measured at λ = 710 nm. The TPC value was expressed as milligrams of gallic acid equivalent (GAE) per gram of dry *S. europaea* stems (mg GAE/g). The results are presented as the mean ± standard deviation (SD), derived from triplicate analyses (*n* = 3).

### 3.8. Determination of Radical Scavenging Activity (DPPH Assay)

The free radical scavenging capacity of each extract was spectrophotometrically evaluated using the Tecan Infinite Pro 200 Microplate Reader and 96-well microtiter plates (Greiner 96 Flat Bottom Transparent Polystyrene) [60]. In each well, 162.5 µL of DPPH solution (0.1 mM in MeOH) freshly prepared was added to 87.5 µL of *S. europaea* extract at different concentrations (0.20–125 µg/mL). The reaction mixture was incubated to react at 30 °C for 30 min. A mixture of 87.5 µL of MeOH and 162.5 µL of DPPH solution was prepared as the control sample. The absorbance of the mixture was estimated spectrophotometrically at λ = 517 nm. The percentage of DPPH radical scavenging activity was calculated by the following equation:

% DPPH radical scavenging activity = (Acontrol − Asample)/Acontrol × 100(1)

where Acontrol is the absorbance of the control, and Asample is the absorbance of the extract.

Antiradical curves were plotted, referring to concentration on the x-axis and % DPPH radical scavenging activity on the y-axis. Then, the IC_50_ concentration necessary for a 50% reduction in the DPPH radical was calculated from the graph. The IC_50_ values are expressed as milligrams of gallic acid equivalent (GAE) per g of dry *S. europaea* stems (mg GAE/g. The experiments were conducted in triplicate at each concentration (*n* = 3). The results are presented as the mean ± standard deviation (SD), derived from triplicate analyses (*n* = 3).

### 3.9. Determination of Antioxidant Activity (ABTS Assay)

The ABTS (ammonium salt 2,2′-azinobis (3-ethylbenzothiazolin-6-sulfonic acid) assay procedure was performed on a microplate reader (Tecan Infinite Pro 200 Microplate Reader) using spectrophotometric detection and 96-well microtiter plates (Greiner 96 Flat Bottom Transparent Polystyrene) [60]. ABTS^+^ solution was fleshly prepared for each assay; the stock solutions (7 mM ABTS^+^ and 2.45 mM K_2_S_2_O_8_) were mixed in equal quantities (1:1) and left to react for 16 h at room temperature in the dark. The solution was then diluted by mixing 1.4 mL ABTS^+^ solution with 30 mL MeOH to obtain the ABTS^+^ working solution. Briefly, the assay was conducted as follows: 10 μL of each extract or standard solution (Trolox at the appropriate concentration) was mixed with 190 μL of ABTS^+^ working solution in a 96-well microplate. A mixture of 10 µL of MeOH and 190 µL of ABTS^+^ working solution was prepared as the control sample. The mixture was then incubated at 37 °C for 10 min before the absorbance was read at λ = 734 nm. ABTS radical scavenging activity was calculated by using the following formula:

ABTS radical scavenging activity = [(Ablank − Asample)/Ablank] × 100(2)

where Ablank is the absorbance of the control, and Asample is the absorbance of the extract. The experiments were repeated in triplicate at each concentration. The calibration curve (y = −3.4395x + 0.7454, R^2^ = 0.999) was found to be linear in the range of 0 to 150 μg/mL of Trolox. The antioxidant activity of each NADES-UAE extract was expressed in mg of Trolox equivalents (TEs) per g of dry *S. europaea* stems (mg TE/g dry). The results are presented as the mean ± standard deviation (SD), derived from triplicate analyses (*n* = 3).

### 3.10. Determination of Ferric Reducing Antioxidant Power (FRAP Assay)

The reducing power of *S. europaea* extracts was evaluated also according to the ferric reducing antioxidant power (FRAP) assay [60]. The FRAP assay procedure was performed on a microplate reader (Tecan Infinite Pro 200 Microplate Reader) using spectrophotometric detection and 96-well microtiter plates (Greiner 96 Flat Bottom Transparent Polystyrene). A FRAP working solution was prepared by mixing three solutions in a 10:1:1 (*v/v/v*) ratio, of which the first contained 20 mmol/L of FeCl_3_, the second 300 mmol/L of acetate buffer (pH 3.6) and the third 10 mmol/L of TPTZ in 40 mmol/L of HCl. In the 96-multiwell plate, 30 μL of sample (*S. europaea* NADES-UAE extracts) or standard solution (Trolox, 7.5–150 μg/mL) was added to 970 μL of the FRAP working solution. The mixture was incubated in darkness for 15 min at 37 °C and then the absorbance was determined at 593 nm. The experiments were repeated in triplicate at each concentration. The calibration curve (y = −0.0128x − 0.0033, R^2^ = 0.9778) was linear in the range of 0 to 150 µg/mL. The reducing power of *S. europaea* extracts were expressed as Trolox equivalent per mg of dry *S. europaea* stems (μg TE/mg dry). The results are presented as the mean ± standard deviation (SD), derived from triplicate analyses (*n* = 3).

### 3.11. Stability of the Extracts

The stability of the extracts was evaluated over a 90-day period. Extracts were stored in the dark at a controlled temperature of 25 ± 2 °C, and TPC was measured at 30, 60 and 90 days, as described in Section 3.6, to monitor changes over time. The results are presented as the mean ± standard deviation (SD), derived from triplicate analyses (*n* = 3).

### 3.12. Antibacterial Activity

The in vitro minimum inhibitory concentrations (MICs, μg/mL) and the minimum bactericidal concentrations (MBCs, μg/mL) were determined by using the broth microdilution method according to the Clinical and Laboratory Standards Institute (CLSI) guidelines [61,62]. A gradient of concentrations ranging from 50% *v*/*v* to 0.05% *v*/*v*, within the wells, was achieved by performing two-fold serial dilutions in the designated testing medium (Mueller–Hinton Broth (Oxoid, Milan, Italy)). We used the following bacterial strains from the American Type Culture Collection (ATCC, Rockville, MD, USA) that were available as freeze-dried discs: *Enterococcus faecalis* 29212 (Gram-positive), *Staphylococcus aureus* 29213 (Gram-positive), *Escherichia coli* 25922 (Gram-negative) and *Klebsiella pneumoniae* 13883 (Gram-negative). Following the CLSI M7-A9 guidelines, the turbidity of the bacterial cell suspension was adjusted to match the 0.5 McFarland Standard using a spectrophotometer (OD625 nm readings between 0.08 and 0.10). This standardized suspension was then diluted further (1:100) using MHB to achieve a concentration of 1–2 × 10^6^ CFU/mL (colony-forming units). Aliquots of 100 μL from the final inoculum were introduced into each well. Two controls were included: medium sterility control (medium without bacterial suspension) and positive control (medium with the suspension of bacteria). Levofloxacin was used as the standard reference antibiotic for this study. To mitigate the risk of interference due to the intrinsic viscosity or coloration of some NADES-based extracts, MIC readings were performed by visual inspection of bacterial growth, as recommended by the CLSI guidelines. No visible interference was observed at the dilutions tested. Additionally, the accuracy of MIC determinations was supported by the subsequent MBC evaluation on agar plates, confirming the bactericidal activity observed in the broth. The plates were incubated at 37 °C for 24 h, with the minimum inhibitory concentration (MIC) values determined as the lowest concentration of the compounds at which no visible bacterial growth was observed [63]. Each experiment was performed three times in duplicate. Levofloxacin was used as the standard reference antibiotic for this study. Moreover, the antibacterial efficacy of each NADES (which served as a solvent for the studied extracts) was assessed in the same range of concentrations of the extracts. To determine the MBC, bacterial cultures treated with the tested compounds at varying concentrations were incubated as described in the MIC assay. Following incubation, 10 μL of each well that showed no visible bacterial growth was plated onto fresh Mueller–Hinton agar plates. The plates were then incubated at 37 °C for 24 h. The MBC was defined as the lowest concentration of the compound at which no visible bacterial growth was observed on the agar plates, indicating that ≥99.9% of the initial bacterial population had been killed. All experiments were performed three times in duplicate to ensure reproducibility.

### 3.13. Data Analysis

All experimental data presented in the text and tables are expressed as the mean ± standard deviation (SD) of the triplicate measurements. The error bars in the figures represent the SD and were calculated using Microsoft Excel. DPPH IC_50_ values were calculated using Graphpad Prism Software, Inc., version 9.5.

Statistical analysis was performed using IBM SPSS Statistics version 25. A *p*-value of <0.05 was considered statistically significant. One-way analysis of variance (ANOVA) was used to assess statistical differences among groups, followed by Tukey’s post hoc test for pairwise comparisons.

Pearson’s correlation coefficients were calculated using Microsoft Excel to compare the activity coefficient ln(γ) of the different NADES extracts with TPC and antioxidant assays (DPPH, ABTS and FRAP) results.

## 4. Conclusions

The NADES-UAE approach investigated in this study has demonstrated it is an effective environmentally friendly solution for the recovery of antioxidant and antimicrobial compounds from autochthonous Apulian *S. europaea* stems, offering a sustainable alternative to conventional solvents, such as 50% EtOH-UAE, used as a control.

Proline:malic acid showed interesting results and could be highlighted as the most promising *S. europaea* NADES-UAE extract based on the antioxidant assays, antimicrobial results and the stability of the TPC values during 90 days of storage.

NADESs being non-toxic and biodegradable and enhancing the extraction of bioactive compounds from non-traditional crops, such as *S. europaea*, could be considered a valuable contribution to green chemistry.

The effectiveness of NADESs in terms of their antioxidant and antimicrobial results as investigated in this study may vary, so it is essential to carefully select the appropriate NADESs and extraction methods for specific applications in pharmaceutical, nutraceutical and cosmeceutical fields.

Further studies using advanced analytical techniques, such as chromatographic and spectrometric analyses, will be needed to fully characterize *S. europaea* NADES extracts to identify bioactive constituents, correlate chemical compositions with bioactivities and explore mechanistic pathways.

## Figures and Tables

**Table 1:** Predicted activity coefficient ln γ solutes for isorhamnetin in NADESs with 30% (*w*/*w*) of water using COSMO-RS.

NADES	Ratio	Ln *γ*	NADES	Ratio	Ln *γ*	NADES	Ratio	Ln *γ*
B:CA	1:1	0.84	ChCl:Gly	1:2	0.39	Fru:Glc:U	1:1:2	2.61
B:Glc	1:1	−0.39	ChCh:Ma	1:1	0.81	Glc:EG	1:2	2.69
B:Gly	1:2	−1.2	ChCh:Mal	4:1	−1.07	Glc:Fru	1:1	2.58
B:OxA:Gly	1:2:1	2	ChCl:OxA	1:1	1.17	Glc:Fru:EG	1:1:2	1.79
B:Ma	1:1	−1.08	ChCl:Pro:Ma	1:1:1	0.26	Gly:Glc	2:1	1.88
B:Ma:Glc	1:1:1	1.01	ChCl:Suc	2:1	1.17	Gly:Sol	2:1	3.13
B:Ma:Pro	1:1:1	−1.1	ChCl:Sol	1:1	1.37	Ma:Fru	1:1	2.51
B:EG	1:1	−3.28	ChCl:Sol	2:3	2.21	Ma:Fru:Gly	1:1:1	2.39
B:EG	1:2	−3.47	ChCl:Sor	1:1	0.54	Ma:Glc	1:1	2.9
B:Arg	1:1	−2.17	ChCl:U	1:2	−1.24	Ma:Glc:Gly	1:1:1	2.7
B:His	1:1	−2.01	ChCl:U:EG	1:2:2	−0.8	Ma:Sor:Gly	1:1:2	2.4
B:Lys	1:1	−3.39	ChCl:U:Gly	1:2:2	0.45	Ma:Suc	2:1	3.82
B:Xyl	1:1	−0.4	ChCl:Xyl	2:1	−1.45	Pro:Glc:Gly	1:1:1	1.58
B:Suc	4:1	−3.88	ChCl:Xyol	5:2	−2.17	Pro:Ma	1:1	1.32
ChCl:CA	2:1	0.46	CA:Fru	1:1	2.95	Suc:EG	1:2	2.99
ChCl:CA	1:1	2.18	CA:Fru:Gly	1:1:1	2.82	Suc:Glc:Fru	1:1:1	3.67
ChCl:EG	1:2	−2.2	CA:Glc	1:1	3.24	Suc:Glc:U	1:1:2	3.92
ChCl:EG	2:1	−4.79	CA:Glc:Gly	1:1:1	3.07	Sol:EG	1:2	2.94
ChCl:Fru	1:1	0.27	CA:Sor	2:3	2.82	Sor:EG	1:2	1.07
ChCl:Glc	2:1	−0.69	CA:Suc	1:1	4.26	Xyl:EG	1:2	1.43
ChCl:Glc	1:1	0.96	Fru:EG	1:2	0.94			

Legend: B—Betaine, CA—Citric Acid, Suc—Sucrose, ChCL—Choline Chloride, U—Urea, Xil—Xylitol, Glc—Glucose, Pro—Proline, MA—Malic Acid, Gly—Glycerol, OxA—Oxalic Acid, Ma—Malic Acid, EG—Ethylene Glycol, Arg—Arginine, His—Histidine, Lys—Lysine, Xyl—Xylose, Mal—Maltose, Sol—Sorbitol, Sor—Sorbose and Fru—Fructose.

**Table 2:** TPC, DPPH, ABTS and FRAP results of *S. europaea* NADES-UAE extracts and EtOH 50% UAE extract. Values are expressed as means ± standard deviation (*n* = 3).

*S. europaea*	TPC (F-C)	DPPH	ABTS	FRAP
NADES	Mg GAE/g	IC_50_ mg GAE/g	Mg TE/g	Mg TE/g
B:CA	2.71 ± 0.004 b	0.17 ± 0.019 b	4.48 ± 0.003 c	1.59 ± 0.008 c,d
B:Suc	2.00 ± 0.001 c	0.27 ± 0.017 c	4.13 ± 0.006 c	1.03 ± 0.002 d
ChCl:U	2.94 ± 0.010 b	0.05 ± 0.009 a	10.96 ± 0.010 a	2.20 ± 0.008 a,b,c
ChCl:Xil	1.48 ± 0.007 d	0.11 ± 0.012 a,b	2.63 ± 0.007 d	0.98 ± 0.008 d
CA:Glc	2.94 ± 0.006 b	0.18 ± 0.023 b	2.23 ± 0.011 d	2.69 ± 0.032 a
Pro:MA	6.79 ± 0.007 a	0.09 ± 0.003 a,b	8.12 ± 0.008 b	2.41 ± 0.017 a,b
EtOH 50%	2.82 ± 0.004 b	0.27 ± 0.005 c	8.65 ± 0.009 b	1.95 ± 0.003 b,c

Different letters within a column indicate significant differences between samples at *p* < 0.05.

**Table 3:** Antibacterial activity (MIC and MBC in % *v*/*v*) of NADESs used in this study.

	*E. faecalis*		*S. aureus* 29213		*E. coli*		*K. pneumoniae* 13883	
	29212				25922			
NADES Solvent	MIC	MBC	MIC	MBC	MIC	MBC	MIC	MBC
B:CA	1.6	3.1	1.6	3.1	1.6	3.1	25	50
B:Suc	50	>50	25	50	>50	>50	>50	>50
ChCl:U	25	50	25	50	>50	>50	>50	>50
ChCl:Xil	12.5	25	12.5	25	25	50	25	50
CA:Glc	1.6	3.1	1.6	3.1	1.6	3.1	3.1	6.3
Pro:MA	6.3	12.5	3.1	6.3	12.5	25	25	50
EtOH 50%	25	50	12.5	25	25	50	25	50

**Table 4:** Antibacterial activity (MIC and MBC in % *v*/*v*) of *S. europaea* NADES-UAE or EtOH 50–UAE extracts and Levofloxacin (µg/mL).

	*E. faecalis* 29212		*S. aureus* 29213		*E. coli* 25922		*K. pneumoniae* 13883	
*S. europaea*	MIC	MBC	MIC	MBC	MIC	MBC	MIC	MBC
NADES-UAE								
B:CA	0.2	0.4	0.2	0.4	0.2	0.4	0.4	0.8
B:Suc	25	50	25	50	50	>50	>50	>50
ChCl:U	25	50	12.5	25	25	50	25	50
ChCl:Xil	25	50	25	50	25	50	>50	>50
CA:Glc	0.2	0.4	0.2	0.4	0.4	0.8	1.6	3.1
Pro:MA	0.1	0.2	0.1	0.2	0.4	0.8	0.4	0.8
EtOH 50%	3.1	6.3	3.1	6.3	12.5	25	25	50
Levofloxacin	2	-	0.5	-	0.12	-	8	-

**Table 5:** Total phenolic content (TPC) monitored over 90 days at 25 °C ± 2 °C.

	TPC mgGAE/g			
*S. europaea* NADES-UAE	T0	T1	T2	T3
B:CA	2.71 ± 0.004	2.81 ± 0.003	2.82 ± 0.006	2.77 ± 0.009
B:Suc	2.00 ± 0.001	1.84 ± 0.005	1.83 ± 0.002	1.29 ± 0.007
ChCl:U	2.94 ± 0.010	2.75 ± 0.002	2.19 ± 0.010	2.03 ± 0.008
ChCl:Xil	1.48 ± 0.007	1.50 ± 0.007	1.58 ± 0.009	1.48 ± 0.015
CA:Glc	2.94 ± 0.006	3.92 ± 0.003	4.38 ± 0.006	4.14 ± 0.012
Pro:MA	6.79 ± 0.007	6.82 ± 0.019	6.86 ± 0.023	6.61 ± 0.010
EtOH 50%	2.82 ± 0.004	2.21 ± 0.013	1.75 ± 0.004	1.80 ± 0.010

Legend: T0 = day 0; T1 = 30 days; T2 = 60 days and T3 = 90 days.

**Table 6:** Composition and acronyms of the studied NADESs.

Hydrogen-Bond Acceptor (HBA)	Hydrogen-Bond Donor (HBD)	Molar Ratio	Acronym
Betaine	Citric Acid	1:1	B:CA
Betaine	Saccarose	4:1	B:Sac
Choline Chloride	Urea	1:2	ChCl:U
Choline chloride	Xylitol	5:2	ChCl:Xil
Citric Acid	Glucose	1:2	CA:Glc
Proline	Malic Acid	1:1	Pro:MA

## Data Availability

Data are contained within the article.
